# Treatment outcomes of penetrating abdominal injury requiring laparotomy at Hiwot Fana Specialized University Hospital, Harar, Ethiopia

**DOI:** 10.3389/fsurg.2022.914778

**Published:** 2022-08-23

**Authors:** Adnan Abdulkadir, Burka Mohammed, Elias Sertse, Melkamu Merid Mengesha, Mathewos Alemu Gebremichael

**Affiliations:** ^1^ School of Medicine, College of Health and Medical Sciences, Haramaya University, Harar, Ethiopia; ^2^ Department of Epidemiology and Biostatistics, School of Public Health, College of Medicine and Health Sciences, Arba Minch University, Arba Minch, Ethiopia; ^3^ Department of Public Health, College of Health Sciences, Bonga University, Bonga, Ethiopia

**Keywords:** penetrating abdominal injury, laparotomy, outcomes, Harar, Ethiopia

## Abstract

**Background:**

Penetrating abdominal injury (PAI) is a public health problem and accounts for significant mortality and disability in both developing and developed countries. It often causes damage to internal organs, resulting in shock and infection. In this study, we assessed the outcomes of PAI and factors associated with post-surgical outcomes including surgical site infection (SSI) and in-hospital death.

**Methods:**

An institution-based cross-sectional study was conducted from 15 January to January 30, 2020, using a standard checklist to review the clinical charts of patients who presented to Hiwot Fana Specialized University Hospital (HFSUH) with PAI and underwent laparotomy between January 2015 and September 2019. Descriptive statistics were used to describe the characteristics of patients, and odds ratios (ORs) with a 95% confidence interval (CI) were reported for factors included in binary logistic regression. The statistical significance was declared at a *P*-value <0.05.

**Results:**

A total of 352 charts of patients with PAI were reviewed. A majority of them (84.9%) were males and the mean age was 26.5 years. The anterior abdomen was the most common site of injury, accounting for 285 patients (81%), 329 patients (93.5%) suffered organ injury, 204 (62%) had a single organ injury, and 125 (38%) had more than one organ injury. The leading injured organs were small intestines 194 (55.1%), followed by the colon 88 (25%) and liver 40 (11.4%). The magnitude of SSI and hospital death was 84 (23.9%) and 12 (3.4%), respectively. Patients above 45 years of age (AOR = 2.9, 95% CI: 1.2, 9.2), with fluid collection (AOR = 2.7, 95% CI: 1.2, 5.9), colostomy (AOR = 3.9, 95% CI: 1.9, 7.8), body temperature >37.5 °C (AOR = 3.8,95% CI:1.9,7.6), and Hgb < 10 mg/dl (AOR = 7.4, 95% CI: 3.4,16.1) had a higher likelihood of SSI. Those patients admitted to the intensive care unit (AOR = 21.3, 95% CI: 1.1, 412.3) and who underwent damage control surgery (AOR = 9.6, 95% CI: 1.3, 73.3) had a higher likelihood of mortality.

**Conclusions:**

SSI and death among patients with PAI were high. Age, fluid collection, colostomy, body temperature, and hemoglobin level were statistically associated with SSI, and intensive care unit and damage control surgery were statistically associated with death. Therefore, health professionals working in surgical wards should consider these factors to alleviate SSI and prevent death. Broadly speaking, the guidelines of the World Society of Emergency Surgery (WSES) should consider these factors in their recommendations.

## Background

According to the 2021 World Health Organization (WHO) report, unintentional and violence-related injuries take the lives of 4.4 million people globally every year and constitute 8% of all deaths (1). Injuries are responsible for an estimated 10% of all years lived with disability, a massive burden on national economies, costing countries billions of US dollars every year in healthcare, lost productivity, and law enforcement (1). Globally, abdominal injury accounts for 25% of all trauma cases, and death due to injury accounts for nearly 1.7 times the number of fatalities that result from HIV/AIDS, tuberculosis, and malaria combined (2, 3).

Penetrating abdominal injury (PAI) is one of the forms of injury that occurs when a foreign object pierces the skin and enters the body, creating a wound. In penetrating trauma, the object remains in the tissue or passes through the tissues and exits the body (4). Penetrating trauma can be caused by violence and may result from fragments of a broken bone, gunshots, and knife wounds (3, 4). Penetrating abdominal trauma often causes damage to internal organs, resulting in shock and infection (4). The most commonly injured organs are the spleen, bowels, stomach, and liver, with the least frequently injured organs being the diaphragm and kidneys (5). However, the severity of the damage depends on the body organs involved, the characteristics of the object, and the amount of energy transmitted (3, 4). PAI is a serious public health problem and among the first reasons for mortality and disability in both developing and developed countries, with significant human, economic, and social costs (3, 4). The mortality rate due to penetrating abdominal trauma varies from 0% to 100% and depends on the organ involved, time to therapy, and how many other organs are involved (4). PAI had been managed conservatively until the early 1900s. However, laparotomy became the standard practice with evidence of a better chance of survival than conservative management (6).

The burden of PAI increases from time to time in both developed and developing countries because of violent crimes and war injuries. It affects 35% of those patients admitted to urban trauma centers and up to 12% of those admitted in suburban or rural centers in the United States (7, 8). A study done in Nigeria Gombe Federal Teaching Hospital reported that penetrating abdominal trauma was seen in a majority (62.9%) of patients (9). In this study, the spleen (29.8%) was the most common isolated injured organ, while the small bowel and the colon (40.7%) were the most injured in combined trauma, and surgical site infection (SSI, 42.9%) was the leading postoperative complication (9). A study from Kenyatta National Hospital revealed approximately 66.2% penetrating abdominal injuries among injured patients (10). In Tanzania, the spleen (75.9%) and gastrointestinal tract (10.3%) were the leading injured organs in patients with a penetrated abdominal injury, approximately 7.8% had a negative laparotomy rate, and the mortality rate was 17.9% (11). In Ethiopia, the burden of PAI out of emergency procedures ranged from 11% to 70% (12). At St. Paul hospital in Addis Ababa, penetrating trauma was the most common injury; stabbing accounted for 35.7% and road traffic accidents (RTAs) 20.9% were the leading causes. In this study, the small intestine (43.8%) and the spleen (34.7%) were the leading injured organs. In this report, common complications were seen in 17.8% of patients, the most common was an irreversible shock (30.4%), and the mortality rate was 8.5% (12).

Despite PAI being one of the leading causes of morbidity and mortality in the developing world, including Ethiopia, little attention has been paid to interventions designed to halt the occurrence of these injuries and the management of the victims. Therefore, this study aimed to assess the treatment outcomes of penetrating abdominal injury and determine the associated factors with poor treatment outcomes requiring laparotomy at Hiwot Fana Specialized University Hospital (HFSUH), Harar, Ethiopia. This will help in designing appropriate management measures for patients with penetrating abdominal injuries.

## Methods

### Study setting and period

The study was conducted in Harar town administration at HFSUH from 15 January to 30 January 2020. Harar town is located in eastern Ethiopia, 525 km away from the capital city Addis Ababa. In Harar town, there are 6 districts and 19 Kebeles (the least administrative unit in Ethiopia) and 2 public health hospitals and 4 health centers. HFSUH is one of the oldest health institutions in the eastern part of Ethiopia. It has a total of 210 beds and approximately 250 health professionals who are serving the community.

### Study design and population

An institution-based cross-sectional study based on the retrospective record review was conducted. All patients who had penetrating abdominal trauma and underwent surgical exploratory laparotomy at HFSUH from January 2015 to September 2019 were included. Those patients who had lost their health cards or had incomplete information on their cards (more than 20% of variables missing) were excluded.

### Sample size determination and sampling technique

All consecutive patients were included in the present study. From logbook registers, all 387 patient cards showing a diagnosis of penetrating abdominal trauma and operation underwent from January 2015 to September 2019 were accepted in this study. Out of all cards, 35 were rejected, and 352 containing complete information were accepted.

### Data collection tool, procedure, and quality assurance

Data were collected by using a standard structured checklist that was developed by reviewing different pieces of literature. The checklist includes socio-demographic characteristics, health profiles of participants, type of injury, operative findings, operations performed, and surgery outcomes. Five and two general practitioners were assigned as data collectors and supervisors, respectively. To assure the quality of data, training was given to data collectors and supervisors. A pretest was done on 5% of the total sample size, and daily supervision was also done.

### Study variables

In this study, the treatment outcome of PAI was a dependent variable. Independent variables were socio-demographic variables such as age, sex, residence, medical/surgical conditions (history), time elapse before presentation to hospital, and clinically related variables such as vital signs before the operation, preoperative hemoglobin, mechanisms of injury, sites of injury, operations performed, and associated injuries.

### Operational definitions

*PAI*: It is an injury penetrating the peritoneum into the abdominal cavity (13).
*Surgical site infection (SSI):* A surgical site infection is present when one of the following criteria is met: purulent discharge from the surgical site, positive culture, the surgical site requires reopening, and SSI is present as judged by the attending physician (12).
*Death*: Patient with PAI admitted and operated at Hiwot Fana Specialized University Hospital and lost his/her life in the hospital during treatment before discharge due to a disease associated with the injury.
*Alive*: PAI victim admitted and started treatment at Hiwot Fana Specialized University Hospital regardless of its cause and discharged cured or without being referred to other hospitals for further treatment (12).

### Data processing and statistical analysis

Before analysis, the completeness and consistency of the data were checked and entered into Epidata software version 4.6. For further management and analysis, data were exported to SPSS window version 22. Descriptive statistics such as proportion, frequency, means, and measure of dispersions were used to describe data. Both bivariable and multivariable binary logistic regression analyses were applied to identify the factors associated with the treatment outcomes of PAI. Those independent variables having *P*-values ≤ 0.25 in the bivariable analysis were entered into the multivariable analysis. In multivariable logistic regression analysis, adjusted odds ratios (AORs) with corresponding 95% confidence intervals were calculated. The statistical significance was declared at a *P*-value of 5%. Hosmer and Lemeshow model fitness test was applied to test model fitness and it was found to be a good fit. Multicollinearity was checked by using the variable inflation factor.

## Results

### Socio-demographic characteristics

A total of 352 records with the complete data were reviewed and included in this study. The majority of 290 (82.4%) of the study subjects were in the age category of 15–45 years, and the mean age was 26.5 ± 11.8 years [standard deviation (SD)]). Males constituted 84.9% of the study subjects and 73.3% of the subjects were rural residents (Table 1).

**Table 1:** Socio-demographic characteristics of study subjects with penetrating abdominal trauma who underwent surgical exploratory laparotomy during 2015–2019 at Hiwot Fana Specialized University Hospital, eastern Ethiopia.

Characteristics	Categories	Frequency (*n*)	Percentage
Age (years)	<15	32	9.1
	15–45	290	82.4
	>45	30	8.5
Sex	Males	299	84.9
	Females	53	15.1
Resident	Urban	94	26.7
	Rural	258	73.3

### Causes of penetrating injury and clinical characteristics

**Figure 1:**
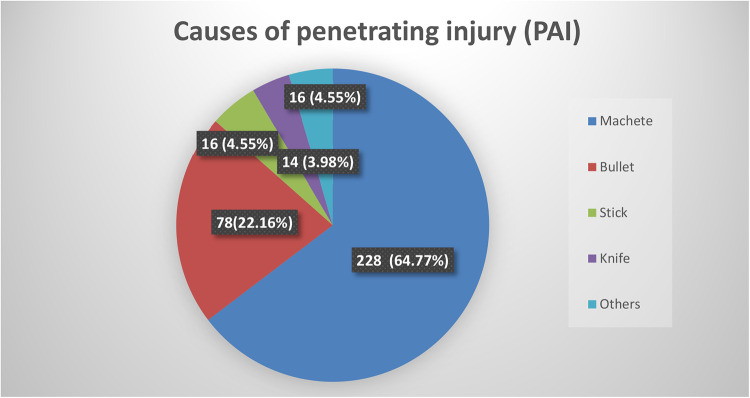
Causes of injury in the study to assess the treatment outcome and pattern of penetrating abdominal trauma victims who underwent surgical exploratory laparotomy during 2015–2019 at HFSUH.

A leading cause of injury was a machete, by which 228 (65%) subjects were victimized, followed by a bullet injury in 78 (22%) (Figure 1). With regard to the mechanisms of injury, stab injuries accounted for 252 (71.6%) of the cases, followed by gunshot 75 (21.3%), blast 8 (2.3%), road traffic accident (RTA) 3 (0.9%), and others. Approximately 258 (73%) of the victims presented within 12 h of injury, whereas 94 (27%) presented to the hospital with a delay of more than 12 h. With regard to vital signs at presentation, systolic blood pressure (SBP) was less than 80 mmHg in 14 (4.0%), pulse rate was greater than 100 in 196 (55.7%), and temperature axillary was <35 °C in 20 (5.7%) patients. The anterior abdomen was the most common site of injury, accounting for 285 (81%), followed by flank 37 (10.5%), back 19 (5.4%), and perineum 11 (3.1%). In the abdominal examination of all victims, 257 (73%) had peritonitis, and evisceration was observed in 80 (22.7%). Associated injuries were seen in 71 (20%) of all patients, and among these, the most common associated injury was chest injury 60 (84.5%), followed by fractures and soft tissue injury 11 (15.5%), and the fluid collection was observed in 171 (48.6%) patients. Of all victims of PAI, 329 (93.5%) patients suffered from organ injury, a majority of 204 (62%) patients had single organ injury, and 125 (38%) had an injury that involved more than one organ. The negative laparotomy (no intra-abdominal organ injury) rate was approximately 6.5%. The leading injured organs were the small intestines 194 (55.1%), followed by colon 88 (25%) and liver 40 (11.4%) irrespective of the mechanism of injury (Table 2).

**Table 2:** Clinical characteristics of patients with PAI in the study to assess the treatment outcome and pattern of penetrating abdominal trauma victims who underwent surgical exploratory laparotomy during 2015–2019 at HFSUH, eastern Ethiopia.

Characteristics	Categories	Frequency (*n*)	Percentage
Temperature (°C)	<35.5	20	5.7
	35.5–37.5	208	64.8
	>37.5	124	35.2
Heart rate (beat/min)	<100	156	44.3
	≥100	196	55.7
Systolic blood pressure (mmHg)	<80	14	4.0
	≥80	338	96.0
Hemoglobin level (mg/dl)	<10	76	21.6
	10–13	213	60.5
	>13	63	17.9
Blood transfusion	Yes	52	14.9
	No	300	84.4
Organ injury	No organ injury	23	6.5
	One organ injury	204	58
	Two organ injuries	97	27.5
	Three or more organ injuries	28	8
Organ injured	Small bowel	194	55.1
	Large bowel	88	25
	Liver	40	11.4
	Gall bladder	9	2.6
	Spleen	22	6.3
	Diaphragm	49	13.9
	Stomach	31	8.8
	Rectum and anal canal	20	5.7
	Renal	11	3.1
	Urinary bladder	8	2.3
	Ureters	2	0.6

### Operative interventions are done for patients with penetrating abdominal injury

Of all victims, repair of hollow or solid organs was done in 200 (56.8%) patients, followed by resection and end-to-end anastomosis in 123 (35%), and damage control surgery (DCS) in 25 (7.1%). Among the total cases, splenectomy was done in 23 (6.5%), 77 (21.9%) underwent colostomy, 24 (6.8%) colectomy, 9 (2.6%) cholecystectomy, 8 (2.3%) bladder repair, and 4 (1.1%) nephrectomy.

### Treatment outcomes and complications

SSI was the most common postoperative complication seen in 84 (23.9%) [95% confidence interval (CI): 19.4–28.3] patients, and a majority 45 (53.6%) of SSI was superficial; deep SSI accounted for 24 (28.6%), and 15 (17.9%) had organ space infections. The second type of complication was pneumonia, which was found in 36 (10.2%) patients. Of all individuals who developed postoperative complications, a total of 22 (6.3%) underwent relaparotomy, and 18 (5.1%) were admitted to the intensive care unit. The mean day of hospital stay in victims who had undergone laparotomy was 6.6 ± 2.4 days (±SD) with minimum and maximum days of stay of 2 and 18, respectively. The magnitude of hospital deaths was found to be 3.4%, and 96.6% were discharged to their homes.

### Factors associated with surgical site infection

In bivariable binary logistic regression analysis, age, causes of trauma, fluid collection, DCS, colostomy, body temperature, heart rate, and hemoglobin were all statistically associated factors with SSI. However, in multivariable binary logistic regression analysis, age, fluid collection, colostomy, body temperature, and hemoglobin were all statistically associated factors (Table 3).

**Table 3:** Bivariable and multivariable binary logistic regression analyses for factors associated with SSI due to penetrating abdominal trauma during 2015–2019 at HFSUH, eastern Ethiopia.

Variables	SSI		COR (95% CI)	AOR (95% CI)
	Yes	No		
Age (years)				
≤45	71	251	Ref.	Ref.
>45	13	17	2.7 (1.3, 5.8)	2.9 (1.2, 9.2)*
Causes of PAI				
Bullet	35	43	3.7 (2.1, 6.5)	1.8 (0.8, 3.8)
Machete	41	187	Ref.	Ref.
Knife	3	11	1.2 (0.3, 4.7)	4.2 (0.7, 24.1)
Stick	3	13	1.1 (0.3, 3.9)	3.7 (0.7, 18.3)
Other	2	14	0.7 (0.1, 2.9)	0.6 (0.1, 3.8)
Fluid collection				
Yes	67	104	6.2 (3.5, 11.2)	2.7 (1.2, 5.9)*
No	17	164	Ref.	Ref.
Damage control surgery				
Yes	13	12	3.9 (1.7, 8.9)	0.6 (0.2, 1.9)
No	71	256	Ref.	Ref.
Colostomy				
Yes	9	15	4.9 (2.8, 8.4)	3.9 (1.9, 7.8)*
No	75	253	Ref.	Ref.
Temperature (°C)				
<35.5	9	11	5.3 (2.0, 13.8)	0.9 (0.2, 3.5)
35.5–37.5	28	180	Ref.	Ref.
>37.5	47	77	3.9 (2.3, 6.7)	3.8 (1.9, 7.6)*
Heart rate (beat/min)				
<100	14	142	Ref.	Ref.
>100	70	126	5.6 (3.0, 10.5)	1.7 (0.8, 3.7)
Hemoglobin (mg/dl)				
<10	41	27	6.5 (3.6, 11.8)	7.4 (3.4, 16.1)*
10–13	42	181	Ref.	Ref.
>13	1	60	0.1 (0.0, 0.5)	0.2 (0.1, 1.2)

AOR, adjusted odds ratio; COR, crude odds ratio; CI, confidence interval; DCS, damage control surgery; PAI, penetrating abdominal injury; Ref., reference category; SSI, surgical site infection.

* Statistically significant (*P*-value <0.05).

The odds of SSI were 2.9 times higher among patients whose age was above 45 years as compared to their counterparts (AOR = 2.9, 95% CI: 1.2, 9.2). Fluid collection was another factor. The odds of SSI were 2.7 times higher among patients who experienced fluid collection when compared with those who had no fluid collection (AOR = 2.7, 95% CI: 1.2, 5.9). The odds of SSI were also 3.9 times higher among patients who had undergone colostomy as compared to their counterparts (AOR = 3.9, 95% CI: 1.9, 7.8). Vital signs at presentation had also a significant association with SSI. The odds of SSI were 3.8 times higher among patients whose body temperature was >37.5 °C as compared to those whose body temperature was between 35.5 °C and 37.5 °C (AOR = 3.8,95% CI:1.9,7.6). Those patients who had Hgb less than 10 mg/dl had 7.4 times higher odds of SSI when compared with those whose Hgb levels were between 10  and 13 mg/dl (AOR = 7.4, 95% CI: 3.4,16.1) (Table 3).

### Factors associated with death due to penetrating abdominal injury

In bivariable binary logistic regression, age, SSI, intensive care unit admission, pneumonia, systolic blood pressure, nephrectomy, colostomy, colectomy, splenectomy, and damage control surgery were statistically associated factors with death due to PAI. In multivariable analysis, intensive care unit admission and damage control surgery were statistically associated factors with death due to PAI (Table 4).

**Table 4:** Bivariable and multivariable binary logistic regression analyses for factors associated with death due to penetrating abdominal trauma during 2015–2019 at HFSUH, eastern Ethiopia.

Variables	Death		COR (95% CI)	AOR (95% CI)
	Yes	No		
Age (years)				
≤45	7	317	Ref.	Ref.
>45	5	23	19.3 (5.7, 65.7)	9.8 (0.8, 119.9)
SSI				
Yes	8	76	7.0 (2.0, 23.7)	17.3 (0.5, 640.1)
No	4	264	Ref.	Ref.
Intensive care unit admission				
Yes	5	13	17.9 (5.0, 64.3)	21.3 (1.1, 412.3)*
No	7	327	Ref.	Ref.
Pneumonia				
Yes	7	29	15.0 (4.5, 50.3)	1.6 (0.1, 29.1)
No	5	311	Ref.	Ref.
Body temperature				
<35.5 °C	8	12	68.7 (13.1, 359.4)	15.3 (0.9, 245.7)
35.5–37.5 °C	2	206	Ref.	Ref.
>37.5 °C	2	122	1.7 (0.2, 12.1)	0.9 (0.1, 21.1)
SBP				
<80 mmHg	4	10	16.5 (4.3, 63.9)	21.4 (0.9, 544.5)
≥80 mmHg	8	330	Ref.	Ref.
Nephrectomy				
Yes	2	2	33.8 (4.3, 264.8)	282.1 (0.2, 399.2)
No	10	338	Ref.	Ref.
Colostomy				
Yes	5	72	2.7 (0.8, 8.6)	2.1 (0.2, 20.0)
No	7	268	Ref.	Ref.
Colectomy				
Yes	3	21	5.1 (1.3, 20.1)	0.2 (0.1, 4.9)
No	9	319	Ref.	Ref.
Splenectomy				
Yes	2	21	3.0 (0.6,14.8)	0.2 (0.0, 279.5)
No	10	319	Ref.	Ref.
Damage control surgery				
Yes	8	17	38 (10.4, 138.8)	9.6 (1.3, 73.3)*
No	4	323	Ref.	Ref.

AOR, adjusted odds ratio; COR, crude odds ratio; CI, confidence interval; Ref., reference category; SSI, surgical site infection; SBP, systolic blood pressure.

* Statistically significant (*P*-value <0.05).

Those patients admitted to the intensive care unit had 21.3 times higher odds of death as compared to those who were not admitted to the intensive care unit (AOR = 21.3, 95% CI: 1.1, 412.3). Patients with PAI who had undergone damage control surgery had 9.6 times higher odds of death when compared with their counterparts (AOR = 9.6, 95% CI: 1.3, 73.3) (Table 4).

## Discussion

In the present study, a stab injury accounted for 252 (71.6%) of the cases, followed by gunshot 75 (21.3%), blast 8 (2.3%), road traffic accident 3 (0.9%), and others. This finding was in agreement with the findings of a study done in Addis Ababa, Ethiopia, where stab was the leading cause of injury, followed by road traffic accidents (12). A study conducted in Tehran, Iran, reported that a majority of injuries were caused by a stab followed by a shot (14).

Abdominal stab and gunshot wounds were far more common in young men than in women, with an overwhelming ratio of 5 men to 1 woman, and the age group below 45 accounted for 85%. As pointed out by other workers in Ghana, the male-to-female ratio was 9:1, and 80% of victims were aged below 45. This is the most productive sector of the population, with serious implications for the national economy and the families that depend on these young traders, farmers, artisans, and businessmen (15). This high percentage in this population might be attributed to engagement in high-risk activities by males and involvement in recreational and risky activities by young age groups who constitute the highly mobile population.

In the present study, of all victims of PAI, 329 (93.5%) had organ injury, with the leading injured organs being small intestines in 194 (55.1%), followed by colon in 88 (25%), liver in 40 (11.4%) and others 7(1.9%). Associated injuries were seen in 71 (20%) of all patients. A study done in Addis Ababa, Ethiopia, revealed that extra-abdominal injuries were seen in 33.3% of the cases. Hollow organs were more commonly injured than solid organs. The small intestine (43.8%) was the leading injured organ (12). Different studies came up with a variety of findings, but a majority of them agreed that the small bowel is a commonly injured organ in penetrating abdominal trauma (9, 10, 16, 17). This might be due to its anatomy being freely mobile and occupying a large area in the abdomen.

The negative laparotomy rate was approximately 6.5% in this setting. This finding was somewhat higher than that in the report on the negative laparotomy rate, which was 4.6%, in a study done in Addis Ababa, Ethiopia (12). This finding was lower than that in other studies done in Kenya, Tanzania, and South Africa, which reported negative laparotomy rates within the range of 7%–16.1% (10, 11, 18). This variation might be due to different kinds of improvements made in patient selection and the availability of staff and facilities. In this study, only patients with penetrating abdominal injuries were included, but other studies such as the one done in Addis Ababa included blunt injury (12).

In the present study, with regard to complications, SSI was a common complication seen in 84 (23.9%) of patients. In other studies done in Addis Ababa, Nigeria, and Tanzania, the irreversible shock was the commonly reported complication (9, 11, 12). The rate of SSI ranged from 13% to 42.9% in different studies (16, 19, 20). This SSI in this study area might be due to the late presentation of patients at the health facility after sustaining the penetrating abdominal trauma. Approximately 94 (27%) presented to the hospital with a delay of more than 12 h, and in the abdominal examination of all victims, peritonitis was revealed in 257 (73%), and evisceration was observed in 80 (22.7%).

The magnitude of hospital death was 3.4% in the present study. This was lower than that in the finding in Addis Ababa, which was 8.5% (12). Other research studies done in Gondar, Kenya, Nigeria, and south-eastern Nigeria showed that the mortality rate in abdominal injury varied from 7.9% to 16.5%, which was higher than that of this study finding (9, 10, 22, 23). This lower mortality rate might be due to improvements made in patient care, availability of qualified staff, and improved facilities in the study area.

From the multivariable binary logistic regression analysis, age was statistically associated with SSI. The odds of SSI were 2.9 times higher among patients whose age was above 45 years as compared to their counterparts. The finding of the present study was in agreement with the report from a study done in Ethiopia on the incidence and predictors of SSI, which reported age as a risk factor for SSI. In this report, patients whose age was greater than 40 years were 7.7 times at higher risk to develop SSI as compared to patients in lower-age groups (23). As the age of the patient’s increases, the likelihood of SSI increases according to the reports of different studies (24). Two possible reasons might be (1) as age increases, the general immunity of a person will decrease, and (2) the occurrence of chronic disease will decrease the immunity of a person, both of which synergistically predispose people to have SSI.

Fluid collection was another factor statistically associated with SSI. The odds of SSI were 2.7 times higher among patients who experienced fluid collection when compared with those who had no fluid collection. The possible explanation for this might be that fluid collection increases the proliferation of microorganisms such as bacteria, which increases the risk of SSI. The finding of the present study was contrary to the study that reported that there was no association between the presence of subcutaneous fluid collection or its volume and the occurrence of SSI (25). The variation might be due to the difference in the definition of fluid collection. In the previous study, the method of clinically asymptomatic subcutaneous fluid collection was used; however, in the present study, the clinically symptomatic fluid collection method was used.

The odds of SSI were 3.9 times higher among patients who had undergone colostomy as compared to their counterparts. This might be due to an entrance of microorganisms during the opening of the colon, or large intestine, through the abdomen, which increases the likelihood of infection as a complication.

Vital signs at presentation also had a significant association with SSI. The odds of SSI were 3.8 times higher among patients whose body temperature was >37.5 °C as compared to those whose temperature was between 35.5 and 37.5 °C. This finding was contrary to the finding of a study done elsewhere (26). This might be due to the delay of patients at presentation in hospital in the present study; approximately 94 (27%) presented to hospital with a delay of more than 12 h. Recent healthcare guidelines for SSI prevention recommend that body temperature be maintained between 35.5 and 36 °C during the perioperative period (27). Therefore, more emphasis should be laid on thermo-regulation before operation in this study setting.

Hemoglobin level is another factor statistically associated with SSI. Among those patients who had hemoglobin levels less than 10 mg/dl had 7.4 times higher odds of SSI when compared with those whose hemoglobin levels were between 10 and 13 mg/dl. Different studies reported that low hemoglobin level (anemia) is a risk factor for SSI (28–31). The possible reason might be that low hemoglobin concentration reduces the oxygen tension in the wound site and increases the risk of SSI by compromising the activity of blood cells such as macrophages and delaying the process of infection healing (32, 33)

Those patients admitted to the intensive care unit had 21.3 times higher odds of death as compared to those who were not admitted to the intensive care unit. In the guidelines of the World Society of Emergency Surgery (WSES), admission to the ICU is recommended for the management of penetrating abdominal trauma (34). However, in the present study, ICU admission was found as a risk factor. The possible reasons could be the severity of the disease and a status standardized ICU. Hence, we can further minimize mortality rates by having a standardized ICU, advanced imaging facilities, and adding a thermoregulatory facility in the ICU as well as an operative room and blood gas analysis unit that will greatly facilitate severe trauma case management.

Patients with PAI who had undergone damage control surgery had 9.6 times higher odds of death when compared with their counterparts. In the guidelines of the WSES, damage control surgery is recommended in the management of patients with penetrating abdominal trauma (34). However, in the present study, damage control surgery was found as a risk factor. The initial aim of damage control surgery was primarily to reduce mortality in exsanguinating patients with coagulopathy. In this surgery, restoring normal physiology took precedence over restoring normal anatomy in unstable, trauma patients to facilitate surgical control of hemorrhage and contamination, stabilization of potentially fatal problems at first-look laparotomy, with secondary resuscitation followed by scheduled definitive surgery (35). Regardless of its primary benefits, in this study setting, damage control surgery was shown as a risk factor for mortality. It was also reported in a previous study that complications and mortality were high in patients undergoing damage control surgery (36). Complications such as wound infection rate (50%–100%), intraabdominal abscess (25%–8%), enterocutaneous fistula (20%–25%), abdominal hypertension in 20% of patients, and mortality (12%–67%) were reported in various studies (37–39) Therefore, the trauma surgeon should follow the recommended guidelines for the application of damage control surgery to minimize mortality rates.

## Conclusions

SSI was found to be high, whereas mortality due to PAI was low in the present study setting. Patient age, fluid collection, colostomy, body temperature, and hemoglobin level were statistically associated with SSI. However, intensive care unit admission and damage control surgery were statistically associated with mortality rates. Therefore, health professionals working in surgical wards should take these factors into the consideration to alleviate SSI and prevent death due to PAI. Broadly speaking, the guidelines that recommend the management of penetrating abdominal trauma, such as WSES, should consider the risk of intensive care unit admission and damage control surgery on vis-à-vis the mortality rate of patients with penetrating abdominal injury when recommending treatment for penetrating abdominal trauma.

## Data Availability

The raw data supporting the conclusions of this article will be made available by the authors without undue reservation.
